# Hypertriglyceridemic waist phenotype and associated factors in individuals with arterial hypertension and/or diabetes mellitus

**DOI:** 10.1017/jns.2021.71

**Published:** 2021-09-14

**Authors:** Luiza Delazari Borges, Luma de Oliveira Comini, Laura Camargo de Oliveira, Heloísa Helena Dias, Emily de Souza Ferreira, Clara Regina Santos Batistelli, Glauce Dias da Costa, Tiago Ricardo Moreira, Rodrigo Gomes da Silva, Rosângela Minardi Mitre Cotta

**Affiliations:** 1 Department of Nutrition and Health, Federal University of Viçosa, Viçosa, Minas Gerais, Brazil; 2 Departament of Nursing and Medicine, Federal University of Viçosa, Viçosa, Minas Gerais, Brazil; 3 Clinical Director of the Hemodialysis Service, São João Batista Hospital, Viçosa, Minas Gerais, Brazil

**Keywords:** Arterial hypertension, Cardiovascular disease, Diabetes mellitus, Hypertriglyceridemic waist phenotype, Primary health care

## Abstract

Cardiovascular diseases are among the main causes of death in Brazil and worldwide. The literature indicates the hypertriglyceridemic waist phenotype (HTWP) as an accessible alternative for the identification of cardiovascular and metabolic risk. The present study aimed to identify the prevalence and factors associated with HTWP in individuals diagnosed with arterial hypertension (AH) and/or diabetes mellitus type 2 (DM2). A cross-sectional study was conducted with individuals diagnosed with AH and/or DM2. The study data were collected through semi-structured interviews containing socio-demographic information, lifestyle, health care, in addition to anthropometric assessment, blood pressure measurement and biochemical blood tests. The prevalence of HTWP was estimated and bivariate and multivariate logistic regression was used to assess the factors associated with HTWP. Of the 788 individuals analysed, 21⋅5 % had the HTWP. In the adjusted model, the following variables remained associated with a greater chance of presenting HTWP: sex, age, body mass index (BMI) and very-low-density lipoprotein (VLDL). Being female increased the chance of HTWP by 7⋅7 times (OR 7⋅7; 95 % CI 3⋅9, 15⋅2). The one-year increase in age increased the chance of HTWP by 4 % (OR 1⋅04; 95 % CI 1⋅02, 1⋅06). The addition of 1 mg/dl of VLDL-c increased the chance of HTWP by 15 % (odds ratio (OR) 1⋅15; 95 % confidence interval (CI) 1⋅12, 1⋅18), as well as the increase of 1 kg/m^2^ in the BMI increased the chance of this condition by 20 % (OR 1⋅20; 95 % CI 1⋅15, 1⋅27). The prevalence of HTWP was associated with females, older age, higher BMI, higher VLDL-c and risk waist/height ratio.

## Introduction

Chronic non-communicable diseases (NCDs), especially cardiovascular diseases (CVDs), are the biggest cause of death in the world, causing approximately 31 % of all deaths globally. About the 17 million premature deaths caused by NCDs, 82 % occur in low- and middle-income countries, 37 % due to CVD. In Brazil, 31⋅2 % of deaths are caused by CVD^(1)^.

Presently, for the diagnosis of abdominal adiposity, the waist–height ratio (WHR), and the waist perimeter (WP) is considered the best risk indicators for CVD^(2–6)^. The main risk factors for cardiovascular events are obesity, dyslipidemia, smoking, physical inactivity, high blood pressure and diabetes mellitus. As the risk factors are associated and added to each other, the possibility of the occurrence of cardiovascular events changes^(7–9)^.

Thus, Lemieux and collaborators in 2000, proposed the hypertriglyceridemic waist phenotype (HTWP), which takes into account the simultaneous increase in WP and triglyceride levels, as an indicator for the identification of cardiovascular and metabolic risk^(10,11)^. It is a low-cost method and easily applicable to the clinic and public health, presenting sensitivity and specificity to track individuals prone to develop CVD^(10,11)^. Studies point out that the hypertriglyceridemic waist represents a discriminating phenotype to identify individuals characterised by an altered cardiovascular and metabolic risk profile^(12)^.

The present study aimed to identify the prevalence and factors associated with HTWP in individuals diagnosed with arterial hypertension (AH) and/or diabetes mellitus type 2 (DM2).

## Experimental methods

### Study design

A cross-sectional study, with a quantitative approach, carried out with individuals diagnosed with AH and/or DM2 accompanied by sixteen primary health care (PHC) teams from Viçosa, Minas Gerais, Brazil, a medium-sized municipality (approximately 78 381 inhabitants), according to the Brazilian Institute of Geography and Statistics^(13)^.

### Study participants

The selection of survey participants was made using the two-stage cluster sampling method, considering the population of 6624 hypertensive and/or diabetic individuals registered and monitored by the PHC in 2017^(13)^. The sample was defined considering 50 % expected phenomenon prevalence, 5 % sampling error margin, 50 % conglomerate effect, 10 % refusals and/or losses, 20 % to control confounding factors and 95 % confidence level. The sample calculation was performed in the Statcalc (program of Epi-Info® version 7.2), and resulted in a sample of 840 individuals, corresponding to 12⋅68 % of the total.

Individuals diagnosed with AH and/or DM2 were chosen because they often present cardiovascular risk factors and coexisting metabolic complications, a characteristic condition of the metabolic syndrome.

The inclusion criteria of the present study were that the participants were 18 years old or older, had a diagnosis of AH and/or DM2, and were registered and monitored by the PHC teams. The study excluded individuals who had severe clinical conditions or who needed specialised care, pregnant women, individuals with a history of alcohol abuse and/or other drugs, bedridden, wheelchair users, people who were unable to go to the PHC unit location for data collection, and those who refused to participate in all stages of the study. Of the 840 individuals selected at random, 52 did not participate in all stages of the study, with a final sample of 788 participants.

This study was conducted in accordance with the Norms and Ethical Guidelines of the Resolution of the National Health Council 510/2016 of the Ministry of Health of Brazil and with the Declaration of Helsinki. The Research Ethics Committee of the Federal University of Viçosa under the number 1203173/2015 approved it. After the reading and signing of the Informed Consent Term, all participants were submitted to anamnesis, clinical, laboratory and anthropometric assessments.

### Data collect

Data were collected in the PHC units between August 2017 and April 2018, through anthropometric assessment, blood pressure measurement, biochemical blood tests and a semi-structured interview guide, with socio-demographic (marital status, age, years of study, colour/race and work), clinic (systolic (SBP) and diastolic blood pressure (DBP)), lifestyle (alcohol and tobacco use) and health care information.

The dependent variable was the HTWP, which is characterised by the simultaneous presence of WC, and triacylglycerols increased. WC was measured immediately above the iliac crest, adopting the cut-off point of the National Cholesterol Education Program (NCEP)^(2)^, and of the World Health Organization (WHO)^(14)^, which classifies as inadequate, values ≥88 cm for women and ≥102 cm for men. Triglyceride values ≥150 mg/dl were considered high. The independent variables were age in years, marital status, colour, work, education in years, number of medications used, alcoholism, tobacco, underlying diseases, self-reported infarction and stroke, diagnosed CKD, number of medications used, body mass index (BMI), glycosylated haemoglobin, fasting blood glucose, total cholesterol, HDL-cholesterol, LDL-cholesterol, VLDL-cholesterol, serum phosphorus, serum calcium, SBP and DBP.

The clinical examination included SBP and DBP and anthropometric measurement. Blood pressure was measured and classified according to the procedures recommended by the VII Brazilian Hypertension Guidelines of 2016^(15)^. AH was defined as SBP ≥ 140 mmHg and/or DBP ≥ 90 mmHg and, or current use of antihypertensive medications^(15)^. Trained researchers, using standard protocols and techniques obtained the anthropometric measurements that consisted of weight, height, BMI, hip circumference (HC) and WHR. The weight was obtained using an electronic scale, with a capacity of 150 kg and a division of 50 g; height was measured using a portable anthropometer, consisting of a metal platform for positioning individuals and a removable wooden column containing millimeter tape and a reading cursor, according to the techniques proposed by Jellife^(16)^. BMI was calculated using the relationship between weight and height squared (P/E^2^). The WC was measured using an inextensible tape and measured in centimetres.

### Statistical analysis

To characterise the study population regarding the variables under study, a descriptive analysis was performed. The normality of the distribution of the continuous variables was tested using the Kolmogorov–Smirnov test. The prevalence of HTWP was estimated, and its association with the characteristics of the individuals was investigated using the *χ*^2^ test for categorical variables and the parametric test (Student's *t*) or non-parametric test (Mann–Whitney) for continuous variables according to the result normality test. For all tests, the significance level was set at 95 %.

The strength of the association between the HTWP and the explanatory variables was assessed using the odds ratio and their respective 95 % confidence intervals using bivariate and multivariate logistic regression.

In the multivariate analysis, the adjusted analysis method used was backward elimination due to likelihood (Wald test). In this sense, all variables that lost their significance were removed from the model one at a time as they did not present significance in the adjustment. Only variables with *P* < 0⋅10 remained in the adjusted model. All analyses were performed using the SPSS program (Statistical Package for Social Science, version 22; SPSS Inc., Chicago, USA).

## Results

Of the 788 individuals analysed, 62⋅7 % were female, 62⋅7 % were married and 43⋅9 % were self-declared brown, yellow or indigenous. The median age was 62 years. Regarding lifestyle habits, 11⋅7 % were smokers, and 27⋅8 % used alcohol. Most individuals reported never having suffered a heart attack (94⋅3 %), stroke (93⋅5 %) and 84⋅5 % reported the presence of CKD. Among the basic diseases, 36⋅3 % have AH and DM2, 55⋅8 % only AH and 7⋅9 % DM2. Other characteristics of interests are presented in Table 1.

**Table 1.** Descriptive and univariate analysis of socio-demographic, clinical, anthropometric and lifestyle habits associated with HTWP

	Total population		Hypertriglyceridemic waist phenotype				
			Not		Yes		
	*n*	%	*n*	%	*n*	%	*P*-value
Sex							0⋅00*
Male	294	37⋅30	264	89⋅79	30	10⋅20	
Female	494	62⋅70	355	71⋅86	139	28⋅13	
Civil State							0⋅571
Single	78	10⋅40	65	83⋅33	13	16⋅66	
Married/friendly	470	62⋅70	373	79⋅36	97	20⋅63	
Separated/divorced	71	9⋅50	53	74⋅64	18	25⋅35	
Widower	131	17⋅50	101	77⋅09	30	22⋅90	
Colour							0⋅009*
Black	173	23⋅30	151	87⋅28	22	12⋅71	
Brown/yellow/indigenous	325	43⋅90	248	76⋅30	77	23⋅69	
White	243	32⋅80	186	76⋅54	57	23⋅45	
Work							0⋅12
Formal	106	13⋅50	88	83⋅01	18	16⋅98	
Informal/Rural	97	12⋅30	83	85⋅56	14	14⋅43	
From home	142	18⋅00	103	72⋅53	39	27⋅46	
Retired	388	49⋅20	303	78⋅09	85	21⋅90	
Unemployed	55	7⋅00	42	76⋅36	13	23⋅63	
Tobacco							0⋅142
Smoker	86	11⋅70	66	76⋅74	20	23⋅25	
Ex-smoker	218	29⋅70	182	83⋅48	36	16⋅51	
Never smoked	431	58⋅60	332	77⋅03	99	22⋅96	
Use of alcohol							0⋅278
Not	532	72⋅20	414	77⋅81	118	22⋅18	
Yes	205	27⋅80	167	81⋅46	38	18⋅53	
Previous diseases							0⋅011*
Arterial hypertension	440	55⋅80	360	81⋅81	80	18⋅18	
Diabetes mellitus type 2	62	7⋅90	51	82⋅25	11	17⋅74	
Hypertension and Diabetes	286	36⋅30	208	72⋅72	78	27⋅28	
Heart attack							0⋅722
Not	693	94⋅30	545	78⋅64	148	21⋅35	
Yes	42	5⋅70	34	80⋅95	8	19⋅04	
Stroke							0⋅666
Not	692	93⋅50	544	78⋅61	148	21⋅38	
Yes	48	6⋅50	39	81⋅25	9	18⋅75	
Chronic kidney disease							0⋅003*
Not	661	84⋅50	533	80⋅63	128	19⋅36	
Yes	121	15⋅50	83	68⋅59	38	31⋅40	

HTWP, hypertriglyceridemic waist phenotype.

* Statistically significant results.

The prevalence of HTWP found was 21⋅5 % (95 % CI), being higher in women (82⋅2 %), in individuals of brown, yellow or indigenous colour, who presented hypertension, DM2 and CKD (Table 1). The prevalence was also higher in participants who use more medications and have higher values of BMI, glycosylated haemoglobin, fasting glucose, total cholesterol, VLDL, calcium, phosphorus and SBP. In the group with HTWP, HDL was lower (Table 2).

**Table 2.** Descriptive and univariate analysis of socio-demographic, clinical, anthropometric, lifestyle and biochemical characteristics associated with HTWP

		Hypertriglyceridemic waist phenotype		
	Total population	Not	Yes	*P*-value
Age^a^	62 (54–69)	62⋅00 (54–69)	64⋅00 (55–69)	0⋅20
Schooling (years of study)^a^	4 (3–6)	4⋅00 (2–6)	4 (3–7)	0⋅49
Number of diseases^a^	0 (0–1)	0 (0–1)	0 (0–1)	0⋅20
Number of medicines^a^	3 (1–4)	2 (1–4)	3(1–5)	0⋅005*
Body mass index^a^	28⋅3 (25⋅2–32)	27⋅3 (24⋅3–30⋅6)	31⋅9 (29⋅6–34⋅6)	0⋅00*
Glycosylated haemoglobin^a^	6 (5⋅6–7⋅1)	6(5⋅6–6⋅9)	6⋅20 (5⋅8–7⋅5)	0⋅002*
Glucose^a^	98 (88–129)	96⋅00 (87–125)	109 (93–143)	0⋅00*
Total cholesterol^b^	191 (40⋅5)	188⋅8 (38⋅9)	199⋅2 (45⋅2)	0⋅003*
HDL-c^a^	49 (41–59)	50 (43–61)	43 (37–51)	0⋅00*
LDL-c^a^	107 (87⋅2–132⋅6)	108 (87⋅8–132⋅4)	103 (84⋅6–133⋅4)	0⋅30
VLDL-c^a^	25⋅2 (19–34⋅6)	22⋅8 (17⋅6–28)	40⋅2 (34⋅6–48⋅6)	0⋅00*
Serum albumin^b^	4⋅7 (0⋅27)	4⋅5 (0⋅3)	4⋅7 (0⋅25)	0⋅99
Phosphor^a^	3⋅4 (3–3⋅8)	3⋅4 (3–3⋅7)	3⋅5 (3⋅1–3⋅85)	0⋅03*
Calcium^a^	9⋅5 (9⋅3–9⋅8)	9⋅5 (9⋅2–9⋅7)	9⋅5 (9⋅3–9⋅8)	0⋅06
Systolic blood pressure^a^	130 (120–140)	130 (120–140)	130 (123–150)	0⋅02*
Diastolic blood pressure^a^	80 (80–90)	80 (80–90)	80 (80–90)	0⋅24

HDL-c, high-density lipoprotein-cholesterol; HTWP, hypertriglyceridemic waist phenotype; LDL-c, low-density lipoprotein-cholesterol; VLDL-c, very-low-density lipoprotein-cholesterol.

a Median (IR).

b Average (sd).

* Statistically significant results.

In the adjusted model, the following variables remained associated with the HTWP: sex, age, risk WHR, BMI and VLDL. Being female increases the chance of developing HTWP by 7⋅7 times (odds ratio (OR) 7⋅7; 95 % confidence interval (CI) 3⋅9, 15⋅2). Having a risk WHR increases the chance of HTWP by 3⋅8 times (OR 3⋅83; 95 % CI 1⋅94, 7⋅60). The increase of one year in age increased this chance of HTWP by 4 % (OR 1⋅04; 95 % CI 1⋅02, 1⋅06). The addition of 1 mg/dl of VLDL-c increased the chance of HTWP by 15 % (OR 1⋅15; 95 % CI 1⋅12, 1⋅18), as well as the 1 kg/m^2^ increased in BMI, which increased the chance of this condition by 20 % (OR 1⋅20; 95 % CI 1⋅15, 1⋅27) (Table 3).

**Table 3.** Crude and adjusted analysis of socio-demographic, clinical, anthropometric, lifestyle and biochemical factors associated with HTWP

	Gross analysis OR (CI 95 %)	Adjusted analysis OR (CI 95 %)
Sex		
Male	1	1
Female	3⋅45 (2⋅25, 5⋅27)	7⋅702 (3⋅9, 15⋅2)
Colour		
Black	1	
Brown/yellow/indigenous	2⋅13 (1⋅27, 3⋅57)	
White	2⋅10 (1⋅23, 3⋅60)	
Age	1⋅00 (0⋅99, 1⋅02)	1⋅04 (1⋅02, 1⋅063)
Work		
Formal	1	
Informal/rural	0⋅82 (0⋅38, 1⋅76)	
From home	1⋅85 (0⋅98, 3⋅46)	
Retired	1⋅37 (0⋅78, 2⋅40)	
Unemployed	1⋅51 (0⋅68, 3⋅38)	
Tobacco	0⋅86 (0⋅39, 1⋅912)	
Hypertension and Diabetes	1⋅68 (1⋅18, 2⋅40)	
Heart attack	0⋅866 (0⋅393, 1⋅91)	
Chronic kidney disease	1⋅9 (1⋅24, 2⋅93)	
Total cholesterol	1⋅00 (1⋅002, 1⋅010)	
Number of diseases	1⋅19 (0⋅96, 1⋅46)	
Glycosylated haemoglobin	1⋅14 (1⋅04, 1⋅26)	
Glucose	1⋅006 (1⋅003, 1⋅009)	
HDL-c	0⋅95 (0⋅93, 0⋅96)	0⋅98 (0⋅96, 1⋅001)
VLDL-c	1⋅12 (1⋅10, 1⋅14)	1⋅15 (1⋅12, 1⋅18)
Phosphor	1⋅41 (1⋅05, 1⋅93)	
Calcium	0⋅99 (0⋅94, 1⋅05)	
Systolic blood pressure	1⋅01 (1⋅00, 1⋅02)	
Body mass index	1⋅15 (1⋅12, 1⋅19)	1⋅20 (1⋅15, 1⋅27)

Initially, all variables from the crude analysis were included in the multivariate analysis, but only those with *P* < 0⋅10 by the Wald test remained in the model.

CI, Confidence interval; HTWP, hypertriglyceridemic waist phenotype; OR, odds ratio; VLDL-c, very-low-density lipoprotein-cholesterol.

## Discussion

A prevalence of 21⋅5 % of HTWP was found. The presence of HTWP remained associated with females, older age, higher BMI, higher VLDL-c and with risk WHR. The present study points out the HTWP as a viable cardiovascular and metabolic risk indicator option to be inserted in clinical practice, as it only involves the measurement of two simple and low-cost measures.

In the ELSA-Brazil study, the prevalence of HTWP was between 13⋅3 % and 24⋅7 %, according to the classification of the WC used (NCEP or International Diabetes Federation [IDF]), which confirms the present study. The literature shows that regardless of the cut-off point used, which is defined through the location of the WC measurement, the HTWP is associated with cardiovascular and metabolic risk factors^(11)^. In another study carried out with adults and using the NCEP cut-off points, the prevalence of HTWP was 26⋅7 %, a result higher than the present study^(17)^. There were also records of lower prevalence in adult populations, such as that reported in a study in Viçosa-MG of 17⋅32 %^(18)^ and a study in southern Brazil^(19)^ indicating 5⋅9 % and 4⋅5 % among men and women, respectively, with an average age of 23 years. Mendes *et al.*^(20)^ still found a prevalence of 21⋅4 %, a result very similar to the present study, however, its screened population was only obese.

The presence of HTWP has numerous implications, including obesity, which is presently the second leading cause of preventable death in Western countries, consequently, there is an increase in visceral fat^(20)^ due to the high correlation between BMI and WC^(9)^. This fact corroborates with the results of the present study due to the finding of a significant association between increased BMI and the presence of HTWP and also with studies conducted in Brazil^(9,20)^ and with a population-based cohort from China^(21)^, indicating that individuals with HTWP have an accumulation of adiposity global and not just in the abdominal region.

The relationship between obesity, high BMI and changes in lipid metabolism, which, in turn, can result in accumulation of these in the liver, muscle, and in the adipose tissue itself is consolidated in the literature^(9)^. In this way, there is a picture of factors associated with each other that generate damage to the population's health. The results of the present study corroborate this finding by demonstrating the association between an increase in VLDL-c with the chance of presenting HTWP. Similar results were found in other study with adult population in Brazil^(11)^.

In parallel with obesity, visceral adipose tissue and dyslipidemic changes, HTWP can also be associated with the development of CVDs, increased c-reactive protein, increased oxidative stress, insulin resistance and high blood pressure^(9,20–22)^. HTWP is an effective and less invasive method to identify individuals susceptible to developing CVDs^(20)^.

The female sex was associated with HTWP. This result differs from others, which mostly shown that there was no difference between the genders^(11,17–20)^. In a study carried out in South America, more cases of HTWP were observed among men (38⋅1 %) than among women (30⋅3 %). Another study reported that the risk of fatal cardiovascular events increased 4⋅7-fold in postmenopausal women with high levels of triacylglycerols, which corroborates with the present study where the majority of women were in postmenopause. The result of the European Prospective Research on Cancer and Nutrition (EPIC)-Norfolk also indicated a greater association of HTWP with females^(23)^.

Mendes *et al.*^(20)^, when analysing a population in the interior of Brazil, found an association between HTWP and increasing on age (OR 1⋅028; 95 % CI 1⋅006, 1⋅052), the same results were found by Freitas *et al.*^(11)^ (*P* < 0⋅001) and in the present study. These findings are explained by the physiology of aging, which leads to metabolic changes that result in the presence of HTWP^(20)^.

The study's limitations include the type of design and the lack of consensus in the literature regarding the definition of the best method to measure the WC, which is directly linked to the HTWP. Thus, there is a need for further studies on this topic. Another limitation is the non-inclusion of important variables to confirm possible associations with HTWP, such as long-term diet, physical activity and sleep habits. As for the strengths of the study, we highlight the representative sample of the population and the use of a database built from a survey with methodological quality, which guarantees the reliability of the data.

The prevalence of HTWP found in the study was high (21⋅5 %) and was associated with females, older age, higher BMI and higher VLDL-c. HTWP is an easily applicable indicator, so its use should be encouraged in health services to predict and decrease the risk of cardiovascular events. It is important to investigate different indicators of cardiovascular and metabolic risk to insert and execute them in practice. The prevention and early treatment of CVDs must be a priority of health policies aiming at health promotion and disease prevention.
